# Granulocyte colony-stimulating factor use does not impair efficacy of chemoimmunotherapy in advanced non-small cell lung cancer: a real-world retrospective study

**DOI:** 10.3389/fimmu.2026.1886001

**Published:** 2026-08-11

**Authors:** Rui Yang, Handai Xia, Ying Zhang, Xinchun Ma, Xuan Sun, Zixu Wang, Ni Liu, Mingzhen Zhang, Xiuwen Wang, Yanguo Liu

**Affiliations:** 1 Department of Oncology, The Affiliated Hospital of Qingdao University, Qingdao, China; 2 Department of Medical Oncology, Qilu Hospital, Cheeloo College of Medicine, Shandong University, Jinan, Shandong, China; 3 Department of Pathology, School of Basic Medical Sciences, Shandong University, Jinan, Shandong, China; 4 Department of Oncology, Qilu Hospital of Shandong University Dezhou Hospital, Dezhou, China

**Keywords:** granulocyte colony-stimulating factor, immune checkpoint inhibitors, non-small cell lung cancer, prognosis, real-world study

## Abstract

**Introduction:**

Granulocyte colony-stimulating factor (G-CSF) is routinely used to manage chemotherapy-induced neutropenia in patients receiving chemoimmunotherapy for advanced non-small cell lung cancer (NSCLC). However, its potential to promote immunosuppressive tumor-associated neutrophils (TANs) has raised theoretical concerns about compromising the efficacy of immune checkpoint inhibitors (ICIs). We aimed to evaluate the real-world association between G-CSF use and survival outcomes in this setting.

**Methods:**

In this retrospective cohort study, we analyzed data from patients with unresectable, locally advanced or metastatic NSCLC who initiated first- or later-line chemoimmunotherapy at a tertiary academic center between 2018 and 2021. Multivariable Cox regression and inverse probability of treatment weighting (IPTW) were used to adjust for key prognostic confounders.

**Results:**

Among 120 enrolled patients, 57 (47.5%) received G-CSF during treatment. With a median follow-up of 22.6 months, the median progression-free survival (PFS) was 12.9 months in the G-CSF group versus 9.6 months in the non-G-CSF group (hazard ratio [HR] = 0.97, 95% CI: 0.60–1.58; P = 0.92). The median overall survival (OS) was 17.6 versus 15.3 months, respectively (HR = 0.99, 95% CI: 0.50–1.98; P = 0.99). In multivariable analysis, G-CSF administration was not independently associated with PFS (adjusted HR [aHR] = 1.08, 95% CI: 0.60–1.93; P = 0.80) or OS (aHR = 1.96, 95% CI: 0.80–4.78; P = 0.14). IPTW-adjusted analyses confirmed these null associations (PFS: HR = 1.30, P = 0.85; OS: HR = 1.00, P = 0.95).

**Discussion:**

In this real-world cohort, no statistically significant association between G-CSF use and survival outcomes was observed among patients with advanced NSCLC treated with chemoimmunotherapy. Further prospective studies are warranted to better define the potential risks and benefits of G-CSF in this setting, particularly regarding its long-term association with survival outcomes.

## 1 Introduction

Lung cancer persists as the foremost cause of cancer-related mortality worldwide, with non-small cell lung cancer (NSCLC) constituting approximately 85% of cases (1, 2). A majority of patients present with advanced disease, historically associated with a dismal prognosis (3–5). The therapeutic paradigm for driver-negative advanced NSCLC has been revolutionized by immune checkpoint inhibitors (ICIs) (6, 7). Particularly, first-line chemoimmunotherapy has markedly improved long-term outcomes, achieving 5-year overall survival rates of 19.4% in unselected populations and up to 31.9% in patients with high PD-L1 expression (8, 9). Nevertheless, innate and acquired resistance remains prevalent, with objective response rates plateauing around 54-60% for combination regimens, underscoring an urgent need to delineate factors that modulate ICI efficacy (10, 11).

The efficacy of ICIs is critically contingent upon the immune contexture of the tumor microenvironment (TME). A “hot” TME, infiltrated by cytotoxic T cells, favors response, whereas an immunosuppressive or “cold” TME is a cornerstone of resistance (12–14). Beyond adaptive immunity, innate immune cells play a pivotal role. Tumor-associated neutrophils (TANs), one of the most abundant leukocyte populations in the TME, exhibit remarkable functional plasticity (15, 16). They can be educated to adopt an anti-tumor (N1) or a pro-tumor (N2) phenotype in response to cytokine signals within the TME (17–19). In particular, the N2-type TANs are potent mediators of immunosuppression, capable of inhibiting T-cell function through various mechanisms, including arginase release, checkpoint protein expression, and metabolic competition, thereby fostering an immune-excluded state (20). Consequently, understanding the regulators of TAN polarization is crucial for overcoming immunotherapy resistance.

Granulocyte colony-stimulating factor (G-CSF), the principal hematopoietic cytokine governing neutrophil production and mobilization, is a cornerstone of supportive care for the prevention and management of chemotherapy-induced neutropenia (21, 22). However, its biological role extends beyond elevating peripheral neutrophil counts. Preclinical evidence indicates that G-CSF can skew neutrophil differentiation towards an immunosuppressive phenotype and enhance their capacity to suppress T-cell proliferation, suggesting a potential conflict with the goals of immunotherapy (23). This raises a pressing, yet unresolved, clinical dilemma: could the routine administration of G-CSF, aimed at mitigating hematological toxicity, inadvertently undermine the efficacy of standard chemoimmunotherapy in NSCLC (24)? To date, robust clinical data addressing this specific concern remain scarce.

To bridge this critical knowledge gap, we conducted a real-world retrospective cohort study. We aimed to evaluate the association between G-CSF use and survival outcomes in patients with advanced NSCLC receiving ICIs combined with chemotherapy, after rigorously adjusting for key prognostic confounders.

## 2 Methods

### 2.1 Study design and population

We conducted a retrospective cohort study at the Department of Oncology, Qilu Hospital of Shandong University. The study included consecutive adults (aged 18–80 years) with histologically or cytologically confirmed, unresectable stage III or IV (AJCC 8th edition) NSCLC, who initiated treatment with a standard regimen of ICIs combined with platinum-doublet chemotherapy between January 2018 and December 2021. Eligible patients were required to have received at least four cycles of the combined therapy and to have an Eastern Cooperative Oncology Group (ECOG) performance status of 0-2. Key exclusion criteria were: 1) incomplete follow-up data; 2) undocumented G-CSF administration status; 3) a history of concurrent active malignancy.

### 2.2 Data collection and variables

Clinical data were systematically extracted from the hospital’s electronic medical records. The following variables were collected: demographics (age, gender, smoking history), disease characteristics (histology, stage), treatment details (line of therapy, specific ICI and chemotherapy agents, dates), and baseline laboratory values within 30 days prior to the first cycle of chemoimmunotherapy. The latter included absolute neutrophil count (ANC), absolute lymphocyte count (ALC), white blood cell count (WBC), and lactate dehydrogenase (LDH) level.

The neutrophil-to-lymphocyte ratio (NLR) was calculated as ANC/ALC. The derived NLR (dNLR) was calculated as ANC/(WBC – ANC). The Lung Immune Prognostic Index (LIPI) score was determined according to a pretreatment lactated hydrogenase (LDH) greater than the upper limit of normal (ULN) and a pretreatment dNLR greater than 3: good LIPI group (dNLR ≤ 3 and LDH ≤ ULN), intermediate LIPI group (either dNLR>3 or LDH > ULN), and poor LIPI group (dNLR >3 and LDH > ULN) (25). The normal range for peripheral blood neutrophil count was 1.8–6.3 ×10^9^/L, with the ULN defined as 6.3 ×10^9^/L.

G-CSF administration (both short-acting and long-acting formulations), including timing relative to chemotherapy cycles, was recorded. For primary analysis, patients were categorized into a G-CSF group (any use during chemoimmunotherapy) or a non-G-CSF group.

### 2.3 Study endpoints and follow-up

The primary endpoint was progression-free survival (PFS), defined as the time from initiation of chemoimmunotherapy to the first documented disease progression according to RECIST v1.1, or death from any cause, whichever occurred first. The secondary endpoint was overall survival (OS), defined as the time from treatment initiation to death from any cause. Patients without an event were censored at the date of last confirmed contact (via clinical visit or telephone interview). Tumor response was assessed according to the Response Evaluation Criteria in Solid Tumors (RECIST) version 1.1, categorized as complete response (CR), partial response (PR), stable disease (SD), or progressive disease (PD). Responses of CR or PR required confirmation after at least 4 weeks.

### 2.4 Statistical analysis

Baseline characteristics were compared between the G-CSF and non-G-CSF groups. Continuous variables were presented as mean ± standard deviation (SD) or median (interquartile range, IQR), and compared using Student’s t-test or Mann-Whitney U test according to data distribution. Categorical variables were presented as frequencies (%) and compared using the Chi-square or Fisher’s exact test.

Survival curves were estimated using the Kaplan-Meier method and compared with the log-rank test. Univariable and multivariable Cox proportional hazards models were used to calculate hazard ratios (HRs) with 95% confidence intervals (CIs). Variables with P < 0.10 in univariable analysis were included in the multivariable Cox models. In addition, clinically relevant variables, including sex, smoking history, histological subtype, treatment line, and LIPI, were also included. Given the potential correlation among inflammatory indicators, sensitivity analyses were performed using separate multivariable Cox regression models. Baseline neutrophil count, NLR, and LIPI were evaluated individually in Model 1-3, rather than being included simultaneously in the same model.

The optimal cut-off values for continuous variables for survival prediction were determined using time-dependent receiver operating characteristic (ROC) curve analysis. The cut-off values were 3.68 × 10^9^/L for baseline neutrophil count and 2.85 for NLR in the PFS analysis, and 3.81 × 10^9^/L for baseline neutrophil count and 3.13 for NLR in the OS analysis.

To address potential confounding due to non-random G-CSF use, we performed propensity score adjustment using inverse probability of treatment weighting (IPTW). The propensity score was estimated using a logistic regression model including age, sex, smoking status, histological subtype, and treatment line. Patients with missing baseline covariates required for propensity score estimation (n = 7) were excluded, resulting in a final weighted cohort of 113 patients. Balance between groups before and after IPTW was assessed using standardized mean differences (SMD), with an SMD < 0.1 indicating good balance. Survival analyses were then repeated in the IPTW-weighted cohort.

All statistical tests were two-sided, and a P-value < 0.05 was considered significant. Analyses were performed using R software (version 4.1.0; R Foundation for Statistical Computing).

## 3 Results

### 3.1 Patient characteristics

A total of 120 patients with advanced NSCLC receiving chemoimmunotherapy were included in the final analysis. Among them, 57 patients (47.5%) received G-CSF during treatment (G-CSF group), while 63 (52.5%) did not (non-G-CSF group). The baseline clinicopathological characteristics of the entire cohort and the two groups are summarized in **Table 1**.

**Table 1:** Baseline characteristics of patients.

Variables	All patients N=120	G-CSF N=57	non-G-CSF N=63	P-value
**Mean age, years**	65.5 ± 8.1	65.6 ± 7.9	65.5 ± 8.3	0.88
Sex				
Male	99 (82.5)	45 (79.0)	54 (85.7)	0.46
Female	21 (17.5)	12 (21.0)	9 (14.3)	
Smoking status				
Yes	91 (75.8)	42 (73.7)	49 (77.8)	0.76
No	29 (24.2)	15 (26.3)	14 (22.2)	
Histology				
SCC	55 (45.8)	26 (45.6)	29 (46.0)	0.82
Non-SCC	65 (54.2)	31 (54.4)	34 (54.0)	
Lines of therapy				
First-line	91 (75.8)	45 (78.9)	46 (73.0)	0.59
Second-line or later	29 (24.2)	12 (21.1)	17 (27.0)	
Clinical stage^*^				
Locally advanced	35 (29.2)	13 (22.8)	22 (34.9)	0.21
Advanced	85 (70.8)	44 (77.2)	41 (65.1)	
**Neutrophil count** **(×10^9^/L)**	4.49 (3.47, 5.93)	3.99 (3.36,5.58)	4.76 (3.77, 6.14)	0.14
**Lymphocyte count** **(×10^9^/L)**	1.59 (1.26, 1.90)	1.40 (1.20,1.82)	1.61 (1.33, 1.90)	0.24
**NLR**	2.85 (2.10, 3.98)	2.71 (2.06, 3.88)	2.97 (2.23, 4.09)	0.33
LIPI				
Good	50 (48.5)	26 (54.2)	24 (43.6)	0.38
Intermediate/poor	53 (51.5)	22 (45.8)	31 (56.4)	

SCC, squamous cell carcinoma; NLR, neutrophil-to-lymphocyte ratio; LIPI, lung immune prognostic index.

^*^ Locally advanced: stage III; Advanced: stage IV.

Bold values indicate statistically significant results (p < 0.05).

The mean age was 65.5 years, 82.5% were male, and 75.8% had a smoking history. Regarding histology, 45.8% had squamous cell carcinoma and 54.2% had non-squamous cell carcinoma (including adenocarcinoma, sarcomatoid carcinoma, and others). Most patients (75.8%) received chemoimmunotherapy as first-line treatment. The median baseline absolute neutrophil count was 4.49×10^9^/L, and the median baseline absolute lymphocyte count was 1.59×10^9^/L, both well-balanced between the two groups (**Table 1**). The median NLR was 2.85 for the overall cohort. The LIPI could be calculated for 103 patients with complete data: 50 patients (48.5%) were classified in the good group, and 53 patients (51.5%) in the intermediate/poor groups combined. There were no significant differences in the distribution of NLR (P = 0.33) or LIPI categories (P = 0.38) between the G-CSF and non-G-CSF groups.

### 3.2 Association between G-CSF use and survival outcomes

**Figure 1:**
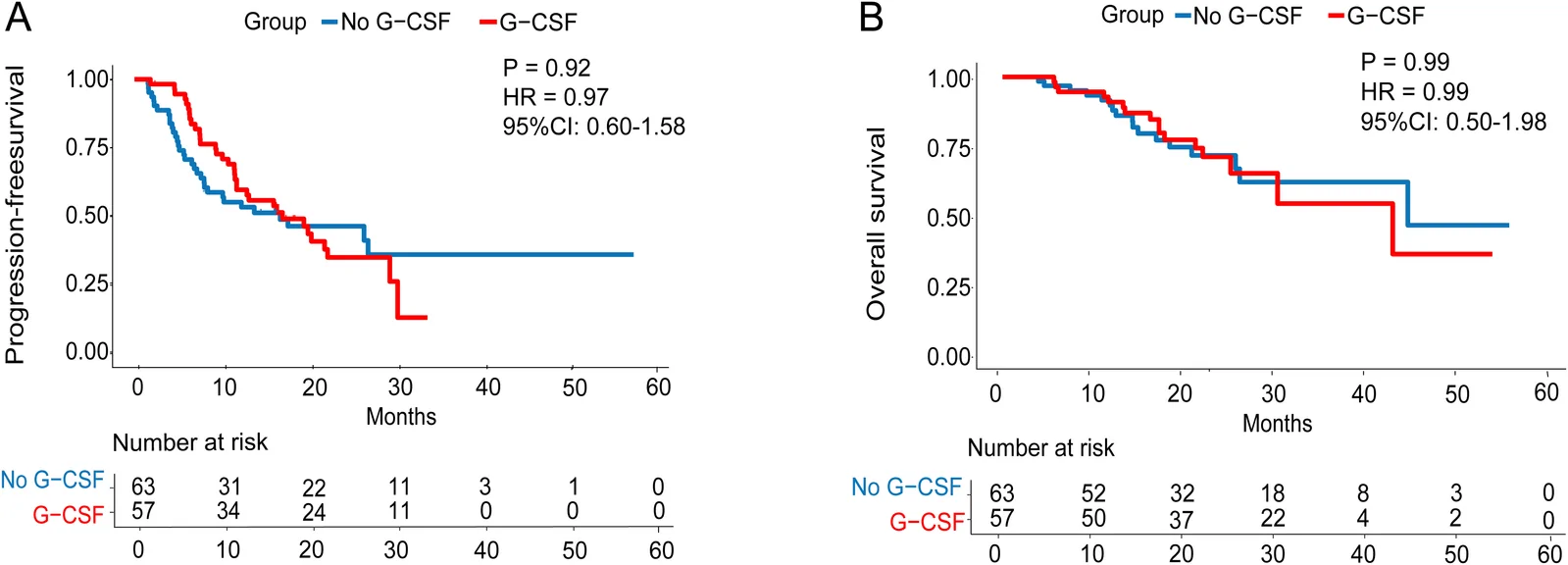
Progression-free survival **(A)** and overall survival **(B)** in all patients, stratified by granulocyte colony-stimulating factor (G-CSF) use. Hazard ratios (HRs) were derived from univariable Cox regression analysis.

With a median follow-up of 22.6 months for the entire cohort, the median PFS was 10.9 months. Patients in the G-CSF group had a median PFS of 12.9 months, compared to 9.6 months in the non-G-CSF group (HR = 0.97, 95% CI: 0.60–1.58; log-rank P = 0.92) (**Figure 1A**). The median OS was 16.0 months overall, 17.6 months in the G-CSF group, and 15.3 months in the non-G-CSF group (HR = 0.99, 95% CI: 0.50–1.98; log-rank P = 0.99) (**Figure 1B**).

**Figure 2:**
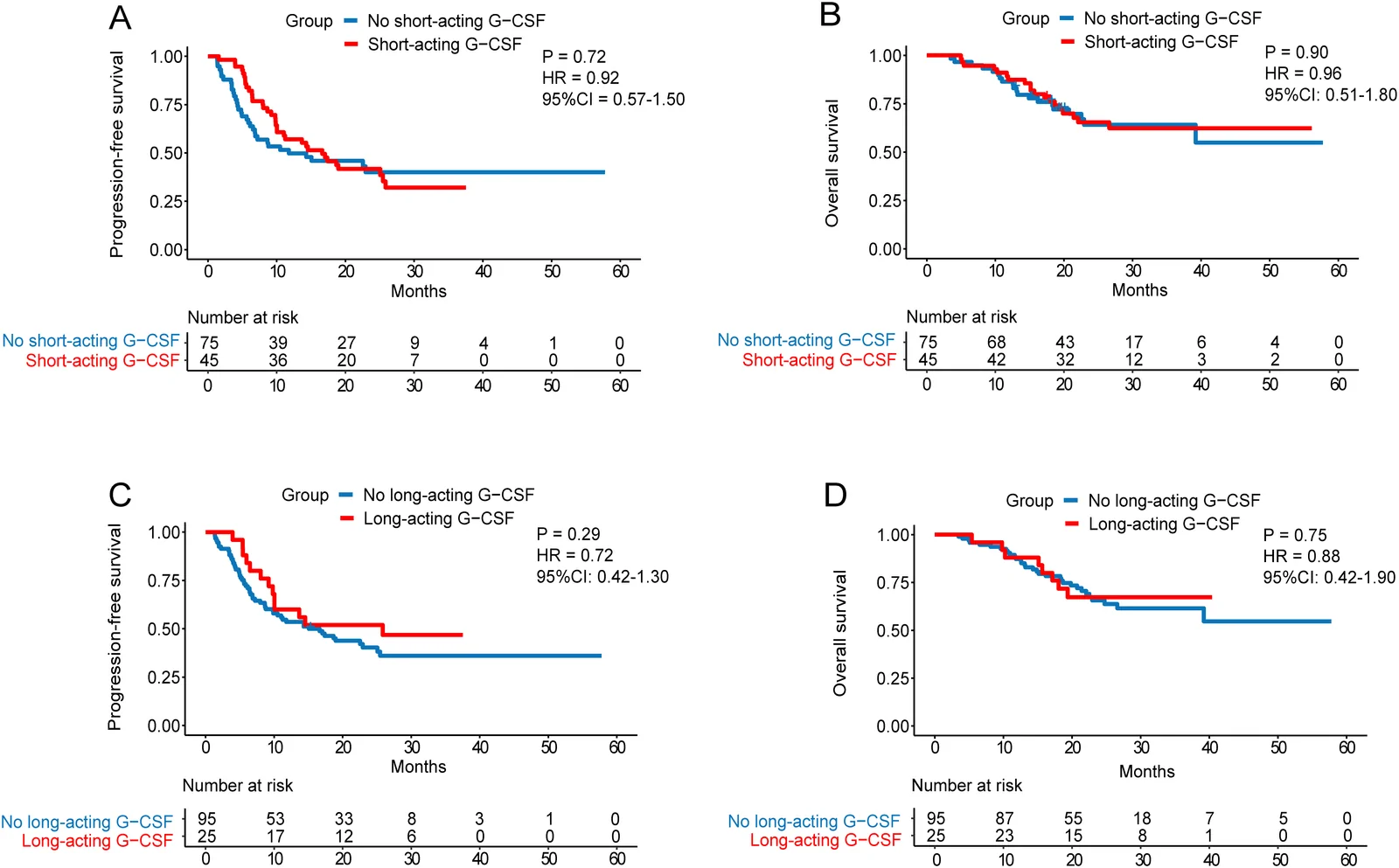
Progression-free survival and overall survival in all patients, stratified by G-CSF formulation. **(A, B)** Short-acting G-CSF versus no short-acting G-CSF. **(C, D)** Long-acting G-CSF versus no long-acting G-CSF. Patients were categorized according to the G-CSF formulation received; patients who received both short-acting and long-acting G-CSF were included in both subgroup analyses. Hazard ratios (HRs) were derived from univariable Cox regression analysis.

**Figure 3:**
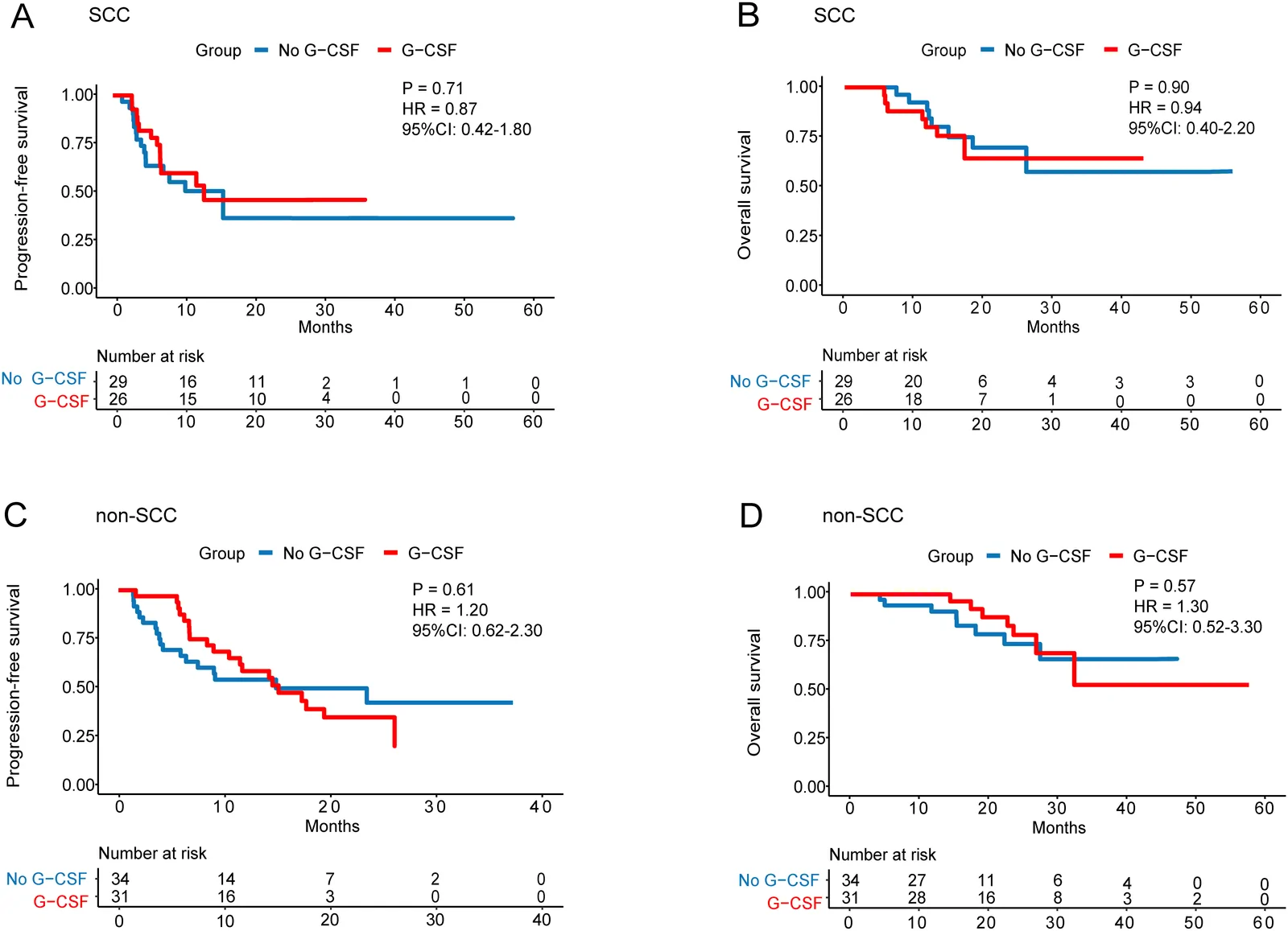
Progression-free survival and overall survival stratified by G-CSF use, according to histology. **(A, B)** Squamous cell carcinoma (SCC). **(C, D)** Non-squamous cell carcinoma (non-SCC). Hazard ratios (HRs) were derived from univariable Cox regression analysis.

**Figure 4:**
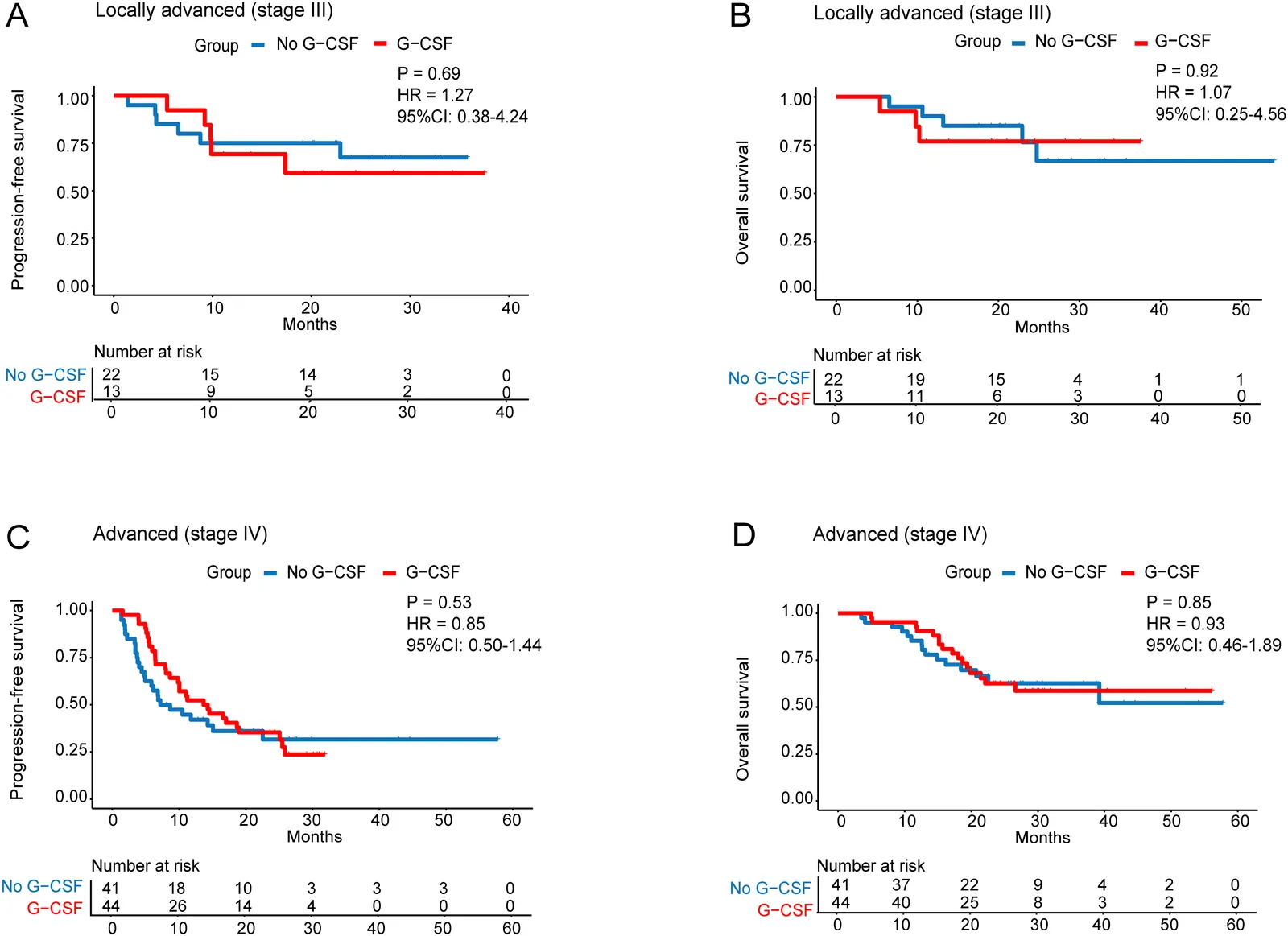
Progression-free survival and overall survival stratified by G-CSF use, according to disease stage. **(A, B)** Locally advanced disease (stage III). **(C, D)** Advanced disease (stage IV). Hazard ratios (HRs) were derived from univariable Cox regression analysis.

Subgroup analyses were performed according to G-CSF formulation. Among G-CSF users, 45 received short-acting and 25 received long-acting formulations. No significant associations between either short-acting or long-acting G-CSF and PFS or OS were observed (all log-rank P > 0.05) (**Figure 2**). We further explored whether the association between G-CSF use and survival outcomes differed across histological subtypes and disease stages. The results showed that G-CSF use was not associated with survival outcomes in either squamous or non-squamous NSCLC patients (**Figure 3**). Similarly, no significant differences in PFS or OS were observed among patients with locally advanced or advanced disease (**Figure 4**).

### 3.3 Univariable and multivariable Cox regression analyses for PFS and OS

The prognostic values of clinical variables for PFS and OS were assessed using Cox regression (**Table 2**). In univariable analysis, advanced disease (HR = 2.51, 95% CI: 1.34–4.69; P < 0.01) and elevated baseline absolute neutrophil count (HR = 2.80, 95% CI: 1.50–5.23; P < 0.01) were significantly associated with shorter PFS. For OS, age > 66 years (HR = 4.20, 95% CI: 1.78–9.93; P < 0.01), elevated baseline absolute neutrophil count (HR = 4.57, 95% CI: 1.73–12.04; P < 0.01), and high NLR (HR = 3.14, 95% CI: 1.46–6.77; P < 0.01) were significantly associated with increased risk of death.

**Table 2:** Univariable Cox regression analyses for PFS and OS.

Variables	PFS		OS	
	HR (95%CI)	P-value	HR (95%CI)	P-value
Mean age, years				
≤ 66	1			
> 66	1.05 (0.64-1.70)	0.86	4.20 (1.78-9.93)	**< 0.01**
Sex				
Male	1		1	
Female	0.84 (0.46-1.55)	0.58	1.16 (0.48-2.82)	0.74
Smoking status				
Yes	1		1	
No	1.03 (0.58-1.81)	0.93	0.65 (0.27-1.58)	0.34
Histology				
SCC	1		1	
Non-SCC	1.23 (0.76-2.00)	0.40	0.71 (0.36-1.41)	0.33
Lines of therapy				
First-line	1		1	
Second-line or later	1.3 (0.76-2.23)	0.34	1.14 (0.52-2.48)	0.74
Clinical stage^*^				
Locally advanced	1		1	
Advanced	2.51 (1.34-4.69)	**< 0.01**	1.38 (0.60-3.20)	0.44
Neutrophil count				
Low	1		1	
High	2.80 (1.50-5.23)	**< 0.01**	4.57 (1.73-12.04)	**< 0.01**
NLR				
Low	1		1	
High	1.47 (0.89-2.43)	0.133	3.14 (1.46-6.77)	**<0.01**
LIPI				
Good	1		1	
Intermediate/poor	1.23 (0.71-2.12)	0.46	1.50 (0.66-3.45)	0.34
G-CSF				
No	1		1	
Yes	0.97 (0.6-1.58)	0.92	0.99 (0.50-1.98)	0.99

PFS, progression-free survival; OS, overall survival; SCC, squamous cell carcinoma; NLR, neutrophil-to-lymphocyte Ratio; LIPI, lung immune prognostic index.

^*^ Locally advanced: stage III; Advanced: stage IV.

Bold values indicate statistically significant results (p < 0.05).

In the multivariable model (**Table 3**), advanced stage [adjusted HR (aHR) = 2.58, 95% CI: 1.21–5.51; P < 0.01] and elevated baseline absolute neutrophil count (aHR = 3.06, 95% CI: 1.29–7.21; P < 0.01) remained independent adverse prognostic factors for PFS. For OS, age > 66 years (aHR = 6.35, 95% CI: 1.81–22.30; P < 0.01) and elevated baseline absolute neutrophil count (aHR = 11.45, 95% CI: 2.83–46.33; P < 0.01) were independently associated with poorer survival. G-CSF use was not independently associated with either PFS or OS in the multivariable analysis.

**Table 3:** Multivariable Cox regression analyses for PFS and OS.

Variables	PFS		OS	
	HR (95%CI)	P-value	HR (95%CI)	P-value
**Mean age, years**			1	
≤ 66	1			
> 66	0.85 (0.46-1.60)	0.60	6.35 (1.81-22.30)	**< 0.01**
Sex				
Male	1		1	
Female	1.06 (0.35-3.21)	0.92	1.43 (0.16-13.00)	0.75
Smoking status				
Yes	1		1	
No	1.01 (0.38-2.73)	0.99	1.14 (0.17-7.84)	0.90
Histology				
SCC	1		1	
Non-SCC	0.98 (0.51-1.90)	0.95	0. 48 (0.17-1.40)	0.18
Lines of therapy				
First-line	1		1	
Second-line or later	1.58 (0.75-3.34)	0.23	1.45 (0.48-4.39)	0.51
Clinical stage^*^				
Locally advanced	1		1	
Advanced	2.58 (1.21-5.51)	**< 0.01**	1.52 (0.54-4.31)	0.43
Neutrophil count				
Low	1		1	
High	3.06 (1.29-7.21)	**< 0.01**	11.45 (2.83-46.33)	**< 0.01**
NLR				
Low	1		1	
High	1.20 (0.61-2.33)	0.59	0.80 (0.28-2.33)	0.69
LIPI				
Good	1		1	
Intermediate/poor	0.89 (0.47-1.70)	0.73	0.97 (0.37-2.58)	0.95
G-CSF				
No	1		1	
Yes	1.08 (0.60-1.93)	0.80	1.96 (0.80-4.78)	0.14

PFS, progression-free survival; OS, overall survival; SCC, squamous cell carcinoma; NLR, neutrophil-to-lymphocyte Ratio; LIPI, lung immune prognostic index.

^*^ Locally advanced: stage III; Advanced: stage IV.

Bold values indicate statistically significant results (p < 0.05).

### 3.4 Inverse probability of treatment weighting

Given the observed imbalances in several baseline characteristics (e.g., gender, histology, treatment line) between the two groups [standardized mean difference (SMD) > 0.1 before adjustment], we performed inverse probability of treatment weighting (IPTW) to minimize confounding (**Table 4**). After IPTW, all baseline covariates were well-balanced (SMD < 0.1 for all).

**Table 4:** Baseline characteristics of patients after IPTW.

Variables	Before IPTW			After IPTW		
	G-CSF N=54	non-G-CSF N=59	SMD	G-CSF N=114.4	non-G-CSF N=111.6	SMD
**Mean age, years**	65.43 (7.76)	65.39 (8.53)	< 0.01	65.16 (7.68)	65.38 (9.04)	0.03
**Sex**			0.13			< 0.01
Male	43 (79.6)	50 (84.7)		95.1 (83.1)	92.8 (83.1)	
Female	11 (20.4)	9 (15.3)		19.3 (16.9)	18.8 (16.9)	
**Smoking status**			0.09			0.02
Yes	40 (74.1)	46 (78)		89.7 (78.4)	86.6 (77.6)	
No	14 (25.9)	13 (22)		24.7 (21.6)	25.0 (22.4)	
**Histology**			0.21			0.07
SCC	25 (46.3%)	28 (47.5)		55.4 (48.4)	53.4 (47.8)	
Non-SCC	29 (53.7%)	31 (52.5)		59.0 (51.6)	58.2 (52.2)	
**Lines of therapy**			0.16			0.04
First-line	43 (79.6)	43 (72.9)		89.8 (78.5)	85.8 (76.9)	
Second-line or later	11 (20.4)	16 (27.1)		24.6 (21.5)	25.8 (23.1)	

SCC, squamous cell carcinoma; IPTW, inverse probability of treatment weighting; SMD, standardized mean difference.

Bold values indicate statistically significant results (p < 0.05).

**Figure 5:**
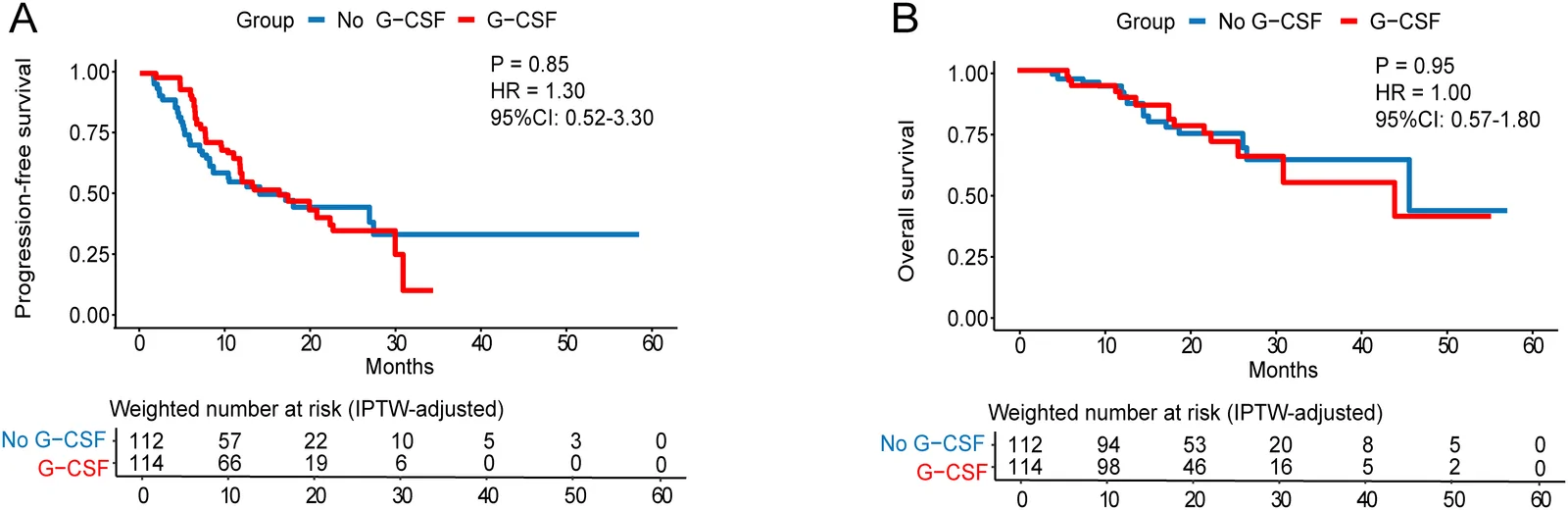
Progression-free survival **(A)** and overall survival **(B)** in all patients after inverse probability of treatment weighting (IPTW) adjustment, stratified by G-CSF use. Hazard ratios (HRs) were derived from IPTW-weighted Cox regression analysis. Risk tables reflect IPTW-weighted risk sets rather than raw patient counts at risk.

In this IPTW-weighted cohort, the association between G-CSF use and survival outcomes remained non-significant for both PFS (HR = 1.30, 95% CI: 0.52–3.30; P = 0.85) and OS (HR = 1.00, 95% CI: 0.57–1.80; P = 0.95) (**Figure 5**).

## 4 Discussion

In this real-world cohort of patients with unresectable NSCLC treated with first-line or later-line chemoimmunotherapy, G-CSF administration for chemotherapy-induced neutropenia was not significantly associated with survival outcomes. After adjustment for key prognostic confounders using multivariable Cox regression and IPTW, we observed no significant difference in either PFS or OS between patients who received G-CSF and those who did not. Similar patterns were observed in subgroup analyses, including stratification by G-CSF formulation (short- vs. long-acting), histological subtype, and disease stage; however, these findings should be interpreted cautiously given the limited sample sizes within individual subgroups. Overall, our study did not observe a statistically significant association between G-CSF use and survival outcomes among patients with unresectable NSCLC treated with chemoimmunotherapy, although prospective studies are warranted for further evaluation.

Our findings address a pivotal translational gap between preclinical concerns and clinical practice. Whereas basic research has consistently demonstrated that TANs can suppress antitumor immunity through multiple mechanisms, raising theoretical concerns that G-CSF might impair immunotherapy by expanding and recruiting TANs (26–28), our clinical results contrast with these preclinical observations, showing no adverse effect of G-CSF on treatment outcomes. Supporting our findings, similar results were reported in a separate multicenter retrospective study involving extensive-stage small cell lung cancer (ES-SCLC) patients treated with a carboplatin/etoposide and atezolizumab regimen, which also observed no significant differences in treatment efficacy between patients who received G-CSF and those who did not (29). Together, these clinical observations suggest that the net immunomodulatory impact of G-CSF in the context of combination chemoimmunotherapy may differ substantially from that inferred from reductionist experimental models.

A potential explanation for our findings lies in the relative magnitude of two distinct forces shaping the systemic and tumor immune milieu: the patient’s inherent, tumor-driven inflammatory state versus the transient, pharmacologic effect of exogenous G-CSF. Our multivariate analysis highlights the dominance of the former. We identified elevated baseline peripheral neutrophil count as a strong, independent adverse prognostic factor for both PFS and OS. This suggests that a pre-existing, cancer-associated neutrophilia is a fundamental determinant of poor outcome, while exogenous G-CSF induces a temporary surge in neutrophil numbers (30, 31).

Within this framework, G-CSF-driven neutrophil expansion may contribute to tumor immune modulation; however, the functional polarization of these recruited neutrophils is likely context-dependent, influenced by the complex cytokine network of the TME which may be altered by the concurrent chemotherapy and ICIs (32–34). In addition, G-CSF also affects both innate and adaptive immunity in preclinical models, primarily via myeloid–lymphoid crosstalk rather than direct lymphocyte targeting (15, 35). T-cell dysfunction is primarily driven by myeloid-derived suppressive activity, mediated through metabolic constraints (e.g., arginase-1–induced arginine depletion) and oxidative stress (36). However, most of these immunomodulatory effects have been demonstrated under conditions of sustained pathway perturbation. In the clinical setting of chemoimmunotherapy for advanced NSCLC, G-CSF is typically administered intermittently and transiently, which may be insufficient to induce durable remodeling of an already established tumor-driven immune ecosystem. Therefore, the profound prognostic impact of the baseline, tumor-elicited inflammatory state may outweigh the subtler and potentially counterbalanced immunomodulatory effects of short-term G-CSF administration.

Collectively, the available clinical evidence—including the present study and reports from other immunotherapy settings—does not suggest a detrimental effect of G-CSF on treatment efficacy when used alongside chemoimmunotherapy. These findings suggest that the biological role of G-CSF in the immunotherapy context is more complex than indicated by preclinical models, and its impact on therapeutic efficacy should be interpreted within the broader clinical setting.

### 4.1 Strengths and limitations

This study has several notable strengths. First, the real-world data used in this study reflects the complexities of clinical practice. Second, rigorous statistical methods were employed, including comprehensive subgroup analysis, multivariate Cox regression, and IPTW, thereby improving the robustness of our findings. Third, this study focuses on a strictly defined and relatively homogeneous patient population, which makes the effect of G-CSF more accurate and reliable. Finally, our work provides important preliminary evidence and hypotheses for conducting larger-scale prospective studies in the future.

However, this study also has several limitations. First, the retrospective design is inherently susceptible to selection bias and residual confounding, despite adjustment using multivariable and propensity-based methods. Second, detailed data on the timing, duration, and cumulative dose of G-CSF were lacking, which may influence the efficacy of immunotherapy and warrants further investigation. Third, potential immortal time bias cannot be fully excluded because the exact timing of G-CSF administration was not consistently recorded in the medical records, which precluded time-dependent or landmark analyses. Fourth, certain variables in the multivariable Cox models were based on limited numbers of events, which may lead to instability of effect estimates. We performed a sensitivity analysis using Firth’s penalized regression to assess the robustness of these findings, which yielded more conservative estimates. Finally, this study lacks in-depth mechanistic exploration. We cannot biologically verify whether and how G-CSF alters TME, particularly the proportion and functional phenotype of TANs, which limits a deeper interpretation of the results.

### 4.2 Conclusion and future perspectives

In conclusion, this real-world retrospective analysis suggests that G-CSF use for the management of neutropenia was not significantly associated with survival outcomes in patients with advanced NSCLC receiving chemoimmunotherapy. However, the clinical implications of G-CSF use in the context of chemoimmunotherapy require further prospective evaluation.

Future efforts should prospectively evaluate these findings in larger-scale, multicenter cohorts with detailed information on G-CSF administration (e.g., timing, duration, cumulative dose). Furthermore, laboratory studies using paired tumor samples before and after G-CSF treatment are needed to clarify its effects on TME, particularly the spatial distribution and functional phenotype of TANs.

## Data Availability

The original contributions presented in the study are included in the article/**Supplementary Material**. Further inquiries can be directed to the corresponding author.
