# Supplementary material for: Granulocyte colony-stimulating factor use does not impair efficacy of chemoimmunotherapy in advanced non-small cell lung cancer: a real-world retrospective study
Source: Front Immunol. 2026 Aug 11;17:1886001. doi: 10.3389/fimmu.2026.1886001 (PMC13503591; doi:10.3389/fimmu.2026.1886001)
Supplement: Supplementary file 1 [file Table1.docx]

Supplementary Table 1. Multivariable Cox regression analyses for PFS and OS using Firth’s penalized likelihood method.

| Variables | PFS | |  | OS | |
| --- | --- | --- | --- | --- | --- |
|  | HR (95%CI) | P-value |  | HR (95%CI) | P-value |
| **Median age, years** |  |  |  | 1 |  |
| ≤ 66 | 1 |  |  |  |  |
| > 66 | 0.99 (0.95–1.03) | 0.70 |  | 1.09 (1.03-1.15) | **< 0.01** |
| **Sex** |  |  |  |  |  |
| Male | 1 |  |  | 1 |  |
| Female | 0.99 (0.31–2.91) | 0.98 |  | 0.63 (0.08-4.61) | 0.66 |
| **Smoking status**  Yes  No |  |  |  |  |  |
| Yes | 1 |  |  | 1 |  |
| No | 1.03 (0.34–2.67) | 0.96 |  | 0.70 (0.09-3.91) | 0.59 |
| **Histology** |  |  |  |  |  |
| SCC | 1 |  |  | 1 |  |
| Non-SCC | 0.92 (0.48–1.76) | 0.81 |  | 0.76 (0.28-1.88) | 0.56 |
| **Lines of therapy**  ≥1 |  |  |  |  |  |
| First-line | 1 |  |  | 1 |  |
| Second-line or later | 2.03 (0.96–4.12) | 0.06 |  | 1.48 (0.51-4.00) | 0.46 |
| **Clinical stage**^*^ |  |  |  |  |  |
| Locally advanced | 1 |  |  | 1 |  |
| Advanced | 2.67 (1.31–5.96) | **< 0.01** |  | 1.42 (0.56-4.25) | 0.48 |
| **Neutrophil count**  counts (×10^9^ ) |  |  |  |  |  |
| Low | 1 |  |  | 1 |  |
| High | 1.23 (1.03–1.47) | **0.03** |  | 1.38 (1.07-1.78) | **0.01** |
| **NLR** |  |  |  |  |  |
| Low | 1 |  |  | 1 |  |
| High | 0.94 (0.73–1.17) | 0.58 |  | 0.92 (0.66-1.23) | 0.59 |
| **LIPI** |  |  |  |  |  |
| Good | 1 |  |  | 1 |  |
| Medium-poor | 0.88 (0.46–1.66) | 0.69 |  | 0.99 (0.38-2.54) | 0.98 |
| **G-CSF** |  |  |  |  |  |
| No | 1 |  |  | 1 |  |
| Yes | 1.14 (0.63–2.02) | 0.66 |  | 1.11 (0.48-2.49) | 0.80 |

**Abbreviations:** PFS, progression-free survival; OS, overall survival; SCC, squamous cell carcinoma; NLR, neutrophil-to-lymphocyte ratio; LIPI, lung immune prognostic index.

^*^Locally advanced: stage III; Advanced: stage IV.

Supplementary Table 2. Multivariable Cox regression analyses of PFS and OS with ULN-based neutrophil grouping.

| Variables | PFS | |  | OS | |
| --- | --- | --- | --- | --- | --- |
|  | HR (95%CI) | P-value |  | HR (95%CI) | P-value |
| **Median age, years** |  |  |  |  |  |
| ≤ 66 | 1 |  |  | 1 |  |
| > 66 | 0.99 (0.95–1.03) | 0.60 |  | 1.09 (1.03–1.16) | **< 0.01** |
| **Sex** |  |  |  |  |  |
| Male | 1 |  |  | 1 |  |
| Female | 0.97 (0.31–2.97) | 0.95 |  | 0.85 (0.11–6.65) | 0.98 |
| **Smoking status**  Yes  No |  |  |  |  |  |
| Yes | 1 |  |  | 1 |  |
| No | 0.92 (0.33–2.60) | 0.87 |  | 0.90 (0.14–5.67) | 0.96 |
| **Histology** |  |  |  |  |  |
| SCC | 1 |  |  | 1 |  |
| Non-SCC | 0.89 (0.46–1.70) | 0.72 |  | 0.74 (0.28–1.99) | 0.81 |
| **Lines of therapy**  ≥1 |  |  |  |  |  |
| First-line | 1 |  |  | 1 |  |
| Second-line or later | 1.88 (0.91–3.85) | 0.09 |  | 1.36 (0.49–3.77) | 0.06 |
| **Clinical stage**^*^ |  |  |  |  |  |
| Locally advanced | 1 |  |  | 1 |  |
| Advanced | 2.66 (1.22–5.82) | **0.01** |  | 1.42 (0.48–4.20) | 0.06 |
| **ULN**^*^ |  |  |  |  |  |
| ≤ULN | 1 |  |  | 1 |  |
| >ULN | 1.78 (0.76–4.15) | 0.19 |  | 4.18(1.24-14.14) | **0.02** |
| **NLR** |  |  |  |  |  |
| Low | 1 |  |  | 1 |  |
| High | 1.03 (0.85–1.26) | 0.74 |  | 0.98 (0.75–1.28) | 0.58 |
| **LIPI** |  |  |  |  |  |
| Good | 1 |  |  | 1 |  |
| Medium-poor | 0.87 (0.45–1.68) | 0.66 |  | 1.12 (0.43–2.93) | 0.69 |
| **G-CSF** |  |  |  |  |  |
| No | 1 |  |  | 1 |  |
| Yes | 0.94 (0.52–1.71) | 0.85 |  | 0.87 (0.37–2.02) | 0.86 |

**Abbreviations:** PFS, progression-free survival; OS, overall survival; SCC, squamous cell carcinoma; ULN, upper limit of normal; NLR, neutrophil-to-lymphocyte ratio; LIPI, lung immune prognostic index.

*Locally advanced: stage III; Advanced: stage IV.

*ULN: neutrophil counts were considered elevated when exceeding 6.3 ×10⁹/L, based on the reference range (1.8–6.3 ×10⁹/L).

Supplementary Table 3. Sensitivity analyses of multivariable Cox regression models using different inflammatory indicators for PFS.

| Variables | Model 1**^*^** | |  | Model 2 | |  | Model 3 | |
| --- | --- | --- | --- | --- | --- | --- | --- | --- |
|  | Neutrophil-adjusted | |  | NLR-adjusted | |  | LIPI-adjusted | |
|  | HR (95%CI) | P-value |  | HR (95%CI) | P-value |  | HR (95%CI) (95%CI) | P-value |
| **Median age, years** |  |  |  |  |  |  |  |  |
| ≤ 66 | 1 |  |  | 1 |  |  | 1 |  |
| > 66 | 0.99 (0.95, 1.02) | 0.48 |  | 0.98 (0.94, 1.02) | 0.27 |  | 0.98 (0.94, 1.02) | 0.38 |
| **Sex** |  |  |  |  |  |  |  |  |
| Male | 1 |  |  | 1 |  |  | 1 |  |
| Female | 0.75 (0.26, 2.18) | 0.60 |  | 0.74 (0.26, 2.12) | 0.57 |  | 0.95 (0.31, 2.87) | 0.92 |
| **Smoking status**  Yes  No |  |  |  |  |  |  |  |  |
| Yes | 1 |  |  | 1 |  |  | 1 |  |
| No | 0.66 (0.24, 1.80) | 0.42 |  | 0.65 (0.24, 1.75) | 0.39 |  | 0.91 (0.32, 2.57) | 0.86 |
| **Histology** |  |  |  |  |  |  |  |  |
| SCC | 1 |  |  | 1 |  |  | 1 |  |
| Non-SCC | 0.85 (0.47, 1.54) | 0.59 |  | 0.79 (0.44, 1.43) | 0.44 |  | 0.75 (0.40, 1.44) | 0.39 |
| **Lines of therapy**  ≥1 |  |  |  |  |  |  |  |  |
| First-line | 1 |  |  | 1 |  |  | 1 |  |
| Second-line or later | 2.00 (1.07, 3.74) | **0.03** |  | 1.91 (1.02, 3.57) | 0.04 |  | 1.87 (0.90, 3.90) | 0.09 |
| **Clinical stage**^*^ |  |  |  |  |  |  |  |  |
| Locally advanced | 1 |  |  | 1 |  |  | 1 |  |
| Advanced | 3.24 (1.60, 6.55) | **< 0.01** |  | 3.12 (1.55, 6.30) | **< 0.01** |  | 2.76 (1.28, 5.95) | **0.01** |
| **G-CSF** |  |  |  |  |  |  |  |  |
| No | 1 |  |  | 1 |  |  | 1 |  |
| Yes | 1.18 (0.69, 2.00) | 0.55 |  | 1.21 (0.71, 2.08) | 0.49 |  | 1.23 (0.68, 2.23) | 0.49 |

**Abbreviations:** PFS, progression-free survival; SCC, squamous cell carcinoma; NLR, neutrophil-to-lymphocyte ratio; LIPI, lung immune prognostic index.

*All models were adjusted for G-CSF use, age, sex, smoking status, histology, treatment line, and clinical stage. Model 1 included baseline neutrophil count, Model 2 included NLR, and Model 3 included LIPI as the inflammatory indicator.

*Locally advanced: stage III; Advanced: stage IV.

Supplementary Table 4. Sensitivity analyses of multivariable Cox regression models using different inflammatory indicators for OS.

| Variables | Model 1**^*^** | |  | Model 2 | |  | Model 3 | |
| --- | --- | --- | --- | --- | --- | --- | --- | --- |
|  | HR (95%CI) | P-value |  | HR (95%CI) | P-value |  | HR (95%CI) (95%CI) | P-value |
| **Median age, years** |  |  |  |  |  |  |  |  |
| ≤ 66 | 1 |  |  | 1 |  |  |  |  |
| > 66 | 1.08 (1.03, 1.15) | 0.09 |  | 1.07 (1.01, 1.13) | **0.02** |  | 1.06 (1.00, 1.13) | **0.04** |
| **Sex** |  |  |  |  |  |  |  |  |
| Male | 1 |  |  | 1 |  |  |  |  |
| Female | 0.24 (0.04, 1.27) | 0.09 |  | 0.29 (0.05, 1.62) | 0.16 |  | 1.02 (0.13, 7.93) | 0.98 |
| **Smoking status**  Yes  No |  |  |  |  |  |  |  |  |
| Yes | 1 |  |  | 1 |  |  |  |  |
| No | 0.32 (0.06, 1.78) | 0.19 |  | 0.44 (0.08, 2.44) | 0.35 |  | 1.08 (0.16, 7.09) | 0.94 |
| **Histology** |  |  |  |  |  |  |  |  |
| SCC | 1 |  |  | 1 |  |  |  |  |
| Non-SCC | 0.65 (0.28, 1.52) | 0.32 |  | 0.60 (0.26, 1.37) | 0.22 |  | 0.55 (0.21, 1.42) | 0.22 |
| **Lines of therapy**  ≥1 |  |  |  |  |  |  |  |  |
| First-line | 1 |  |  | 1 |  |  |  |  |
| Second-line or later | 1.37 (0.56, 3.35) | 0.49 |  | 1.16 (0.47, 2.87) | 0.74 |  | 1.56 (0.57, 4.25) | 0.39 |
| **Clinical stage**^*^ |  |  |  |  |  |  |  |  |
| Locally advanced | 1 |  |  | 1 |  |  |  |  |
| Advanced | 2.17 (0.81, 5.80) | 0.12 |  | 2.08 (0.80, 5.42) | 0.13 |  | 1.70 (0.60, 4.82) | 0.32 |
| **G-CSF** |  |  |  |  |  |  |  |  |
| No | 1 |  |  | 1 |  |  |  |  |
| Yes | 1.14 (0.53, 2.44) | 0.73 |  | 1.25 (0.57, 2.72) | 0.57 |  | 1.17 (0.51, 2.71) | 0.71 |

**Abbreviations:** OS, overall survival; SCC, squamous cell carcinoma; NLR, neutrophil-to-lymphocyte ratio; LIPI, lung immune prognostic index.

*All models were adjusted for G-CSF use, age, sex, smoking status, histology, treatment line, and clinical stage. Model 1 included baseline neutrophil count, Model 2 included NLR, and Model 3 included LIPI as the inflammatory indicator.

*Locally advanced: stage III; Advanced: stage IV.

Supplementary Figure 1. Patient flow diagram of study cohort selection and analysis sets.

Supplementary Figure 2. Progression-free survival (A) and overall survival (B) stratified by baseline neutrophil level according to the upper limit of normal (ULN). Patients were categorized into ≤ULN (within normal range) and >ULN (above normal range).
